# Year Book of Treatment

**Published:** 1890-06

**Authors:** 


THE YEAR-BOOK OF TREATMENT. 1890. Lea Bros., Philadelphia, publishers.

As indicated by the title of the book, it contains many important advances made in treatment of disease during the year 1890. The book consists of twenty chapters, ably edited, comprising in their scope the literature of all countries. The volume, though small, is quite comprehensive, and may be regarded as the exponent of the best contributions, during the past year, in the domain of treatment—medical and surgical.

